# DiffPhys: Enhancing Signal-to-Noise Ratio in Remote Photoplethysmography Signal Using a Diffusion Model Approach

**DOI:** 10.3390/bioengineering11080743

**Published:** 2024-07-23

**Authors:** Shutao Chen, Kwan-Long Wong, Jing-Wei Chin, Tsz-Tai Chan, Richard H. Y. So

**Affiliations:** 1 PanopticAI, Hong Kong Science and Technology Parks, New Territories, Hong Kong, China; schenbq@connect.ust.hk (S.C.); kylewong@panoptic.ai (K.-L.W.); nickchin@panoptic.ai (J.-W.C.); tericchan@panoptic.ai (T.-T.C.); 2 Department of Industrial Engineering and Decision Analytics, The Hong Kong University of Science and Technology, Clear Water Bay, Kowloon, Hong Kong, China

**Keywords:** remote photoplethysmography, deep learning, diffusion model, vital signs measurement

## Abstract

Remote photoplethysmography (rPPG) is an emerging non-contact method for monitoring cardiovascular health based on facial videos. The quality of the captured videos largely determines the efficacy of rPPG in this application. Traditional rPPG techniques, while effective for heart rate (HR) estimation, often produce signals with an inadequate signal-to-noise ratio (SNR) for reliable vital sign measurement due to artifacts like head motion and measurement noise. Another pivotal factor is the overlooking of the inherent properties of signals generated by rPPG (rPPG-signals). To address these limitations, we introduce DiffPhys, a novel deep generative model particularly designed to enhance the SNR of rPPG-signals. DiffPhys leverages the conditional diffusion model to learn the distribution of rPPG-signals and uses a refined reverse process to generate rPPG-signals with a higher SNR. Experimental results demonstrate that DiffPhys elevates the SNR of rPPG-signals across within-database and cross-database scenarios, facilitating the extraction of cardiovascular metrics such as HR and HRV with greater precision. This enhancement allows for more accurate monitoring of health conditions in non-clinical settings.

## 1. Introduction

Photoplethysmography (PPG), introduced in the 1930s [1], provides a non-invasive method for detecting blood volume changes in the microvascular bed of tissue. From the signals generated by PPG (PPG-signals), vital signs such as heart rate (HR), heart rate variability (HRV), and blood oxygen saturation can be measured. Traditional PPG relies on sensors attached to the skin to obtain accurate readings. However, this approach is not suitable for individuals with allergic or sensitive skin, such as newborns. To address these limitations, remote photoplethysmography (rPPG) has been developed as a promising alternative.

RPPG employs webcams to remotely capture facial videos of subjects, detecting and analyzing subtle changes in skin color. The goal of rPPG is to generate signals (rPPG-signals) that closely resemble the corresponding PPG-signals. Traditional rPPG methods involve obtaining RGB time series by averaging pixel values within regions of interest (ROI) and identifying linear subspaces that exhibit the highest optical variations caused by blood flow [2,3,4,5]. With the rapid development of computer technology, deep-learning-based rPPG extraction methods that demonstrate superior performance in rPPG measurement have been proposed [6,7,8,9,10].

Despite advancements, traditional rPPG techniques often yield signals with inadequate signal-to-noise ratio (SNR) due to artifacts like head motion and measurement noise. The SNR, which is the ratio of the power of the desired physiological signal (such as the PPG-signal) to the power of the noise, is crucial for the accurate estimation of vital signs like heart rate (HR) and heart rate variability (HRV). HRV in particular is highly sensitive to SNR as it reflects the adaptability and responsiveness of the cardiovascular system. However, this critical metric often receives insufficient attention in rPPG contexts. Artifacts during signal extraction can significantly degrade the SNR, thereby reducing the accuracy of vital sign estimates. Therefore, advanced algorithms that enhance the SNR of rPPG-signals are essential for PPG to fully realize its potential in HRV estimation.

Moreover, the inherent properties of rPPG-signals, particularly their regular, periodic patterns corresponding to changes in skin color due to the pulsatile nature of blood flow, have been largely overlooked. These periodic properties provide valuable information that can be leveraged to enhance the robustness and SNR of rPPG-signals, but previous work has not adequately utilized this aspect.

To address these limitations, generative models present a promising solution by taking raw rPPG signals as input and producing enhanced versions. Building on this concept, Song et al. [11] introduced PulseGAN, a generative adversarial network (GAN) model designed to generate realistic pulse waveforms. However, GAN-based models face challenges such as mode collapse and training instability, which can degrade the quality of generated rPPG signals. To overcome these issues, we propose DiffPhys, a novel deep generative diffusion model specifically designed to enhance the SNR of rPPG-signals. DiffPhys aims to generate enhanced rPPG-signals that closely resemble the corresponding PPG-signals. DiffPhys consists of a forward process and a reverse process. During training, a distorted version of the target enhanced rPPG-signals is generated, and DiffPhys leverages a neural network to recover the target enhanced rPPG-signals from these distorted signals. With pretraining on a diverse array of PPG-signals, DiffPhys capitalizes on the periodic nature of blood flow, which has been overlooked in previous work, to improve the robustness and SNR of rPPG-signals and enhance the accuracy of HR and HRV estimations.

The paper is organized as follows: Section 2 provides an overview of rPPG technology and generative models. Section 3 details the DiffPhys model structure and the refined reverse process, while Section 4 and Section 5 present experimental setups and results. Section 6 offers a detailed discussion of the results, demonstrating the method’s effectiveness across diverse datasets.

## 2. Related Work

### 2.1. Remote Photoplethysmography Measurement

The field of remote photoplethysmography (rPPG) measurement has significantly evolved since Verkruysse et al.’s early exploration of rPPG-based techniques for assessing physiological parameters [2]. Analytical methods initially employed hand-crafted features and selectively combined information from different color channels. These methods proved effective for enhancing the detection of subtle rPPG-signals [12]. Statistical methods, including Independent Component Analysis (ICA), Principal Component Analysis (PCA), and matrix completion, have provided foundational techniques for decomposing and analyzing physiological signals.

For instance, ICA [3] has been effectively utilized to separate color channels into independent components, with the second component often identified as the rPPG signal for heart rate estimation from facial videos. PCA [13] emphasizes the principal variation component corresponding to the rPPG signal through linear projection. Matrix completion [14] employs self-adaptive matrix completion to identify underlying time-varying factors within a certain range as the rPPG signal from chrominance time series. It has been proved useful for robust heart rate monitoring.

Deep-learning-based methods have further revolutionized rPPG extraction by leveraging the power of convolutional neural networks (CNNs) and attention mechanisms. PhysNet [7] employs a 3D-CNN to directly map facial videos to 1D rPPG-signals, showing better performance than the combination of 2D-CNN and Recurrent Neural Networks (RNNs). DeepPhys [6] combines two branches of CNNs, using spatial and temporal attention to focus on relevant facial regions and track pulsatile signals over time. RhythmNet [8] utilizes CNNs and long short-term memory (LSTM) units to capture temporal dynamics, achieving high HR estimation accuracy. MTTScan [15], a multi-task temporal-spatial attention network, simultaneously learns multiple physiological signals, improving rPPG extraction accuracy and robustness.

PhysFormer [9] introduces a novel transformer-based architecture specifically tailored for rPPG extraction. The model employs a series of multi-head self-attention (MHSA) layers that capture both spatial and temporal dependencies in video data. This architecture contrasts with traditional CNNs by focusing on self-attention mechanisms that dynamically weigh different parts of the input data, enhancing the model’s ability to isolate the rPPG-signals amidst noise and motion artifacts.

Collectively, traditional and deep-learning-based rPPG extraction approaches demonstrate significant improvements in accuracy, robustness, and applicability in diverse environments, underscoring their potential for real-world, non-contact physiological monitoring applications. Nevertheless, previous approaches often overlook the intrinsic characteristics of rPPG-signals, which are crucial for ensuring effective rPPG extraction. Recognizing and leveraging these intrinsic properties is essential for advancing the field of rPPG and improving the accuracy and reliability of non-contact physiological monitoring.

### 2.2. Generative Models

During the training process, generative models learn the distribution of the training data and generate new data by sampling from this distribution. By training with rPPG-signals that exhibit specific patterns, generative models learn to generate signals with similar characteristics, such as periodicity. Therefore, generative models offer an alternative approach to implicitly impose intrinsic patterns on the generated data. In 2014, Ian Goodfellow and colleagues introduced the generative adversarial network (GAN) [16], which demonstrated remarkable success in various domains, including image processing [16] and speech enhancement [17,18]. GANs operate by pitting two neural networks against each other, a generator that creates data and a discriminator that evaluates its authenticity, leading to increasingly realistic outputs through iterative adversarial training.

Building on this concept, Song et al. [11] introduced PulseGAN to enhance the SNR of rPPG-signals. PulseGAN comprises a generator designed to enhance the SNR of rPPG-signals and a discriminator tasked with distinguishing the enhanced rPPG-signals from the reference PPG-signals. By leveraging adversarial training, where the generator and discriminator are trained simultaneously in a competitive setting, PulseGAN significantly improved the SNR of rPPG-signals. This approach resulted in advanced accuracy in the estimation of HR and HRV, demonstrating substantial potential for practical applications.

Despite their effectiveness, GAN-based models encounter notable challenges such as mode collapse, where the generator produces limited varieties of outputs, and training instability, which can lead to difficulties in achieving a balanced training state between the generator and discriminator. These issues can hinder the consistent performance and reliability of the GAN models in practical scenarios.

Diffusion models present a promising alternative by addressing the limitations of GANs. Diffusion models gradually transform simple distributions into complex data distributions through a series of steps, providing a more stable and robust framework for data generation. This method mitigates the risk of mode collapse and enhances training stability, making the diffusion model a viable solution to the problems encountered with GAN-based approaches.

### 2.3. Denoising Diffusion Probabilistic Model

The Denoising Diffusion Probabilistic Model (DDPM), proposed by Ho et al. [19], involves a series of steps that gradually generate data from the target distribution from the initial Gaussian noise. Let $q_{data}\left(x_{0}\right)$ denote the distribution of the target data $x_{0}$ (which is the ground truth PPG in this case) on $R^{L}$, where *L* is the sample length.

The DDPM consists of a forward and a reverse process. In the forward process, the DDPM gradually adds Gaussian noise $\epsilon_{t}∼N\left(0,I\right)$ to the target data $x_{0}$ over a predefined number of *T* steps until the target data are approximately transformed into isotropic Gaussian noise $x_{T}$. The intermediate noisy data are called the latent variable, denoted by $x_{t}$ for t=1,⋯,T. This process is known to be Markovian, and the joint distribution of the latent variables is as follows:

(1) $$q\left(x_{1},\cdots ,x_{T}|x_{0}\right)=\prod_{t=1}^{T}q\left(x_{t}|x_{t-1}\right)$$

where $q\left(x_{t}|x_{t-1}\right)=N\left(x_{t};\sqrt{1-\beta_{t}}x_{t-1},\beta_{t}I\right)$, and $\beta_{t}$ is a predefined small positive constant serving as variance schedule during this process.

The reverse process starts from Gaussian noise $x_{T}$ at step T and gradually eliminates the Gaussian noise added at each step during the forward process to recover the target data $x_{0}$. The reverse process utilizes another Markov chain to define the transition from $x_{T}$ to $x_{0}$:

(2) $$p_{\theta }\left(x_{0},\cdots ,x_{T-1}|x_{T}\right)=\prod_{t=1}^{T}p_{\theta }\left(x_{t-1}|x_{t}\right)$$

where θ parameterizes a neural network to estimate the Gaussian noise added at step t−1, denoted by $\epsilon_{\theta }$, based on the observation of $x_{t}$ for t=1,⋯,T. θ is estimated by maximizing the likelihood of joint distribution $p_{\theta }\left(x_{0},\cdots ,x_{T-1}|x_{T}\right)$.

Although it is intractable to directly calculate $p_{\theta }\left(x_{0},\cdots ,x_{T-1}|x_{T}\right)$, Ho et al. [19] suggested maximizing the probability of the joint distribution by optimizing its variational lower bound, which is equivalent to minimizing the following loss function:

(3) $$L\left(\theta \right)=E_{x_{0},\epsilon ,t}{\left\|\epsilon -\epsilon_{\theta }\left(\sqrt{{\bar{\alpha }}_{t}}x_{0}+\sqrt{1-{\bar{\alpha }}_{t}}\epsilon ,t\right)\right\|}_{2}^{2}$$

where $\alpha_{t}=1-\beta_{t}$, and ${\bar{\alpha }}_{t}=\prod_{i=1}^{t}\alpha_{i}$. After estimation of the added noise ϵθ, the previous step can be sampled according to the following equation:

(4) $$x_{t-1}=\frac{1}{\sqrt{{\bar{\alpha }}_{t}}}\left(x_{t}-\frac{\beta_{t}}{\sqrt{1-{\bar{\alpha }}_{t}}}\epsilon_{\theta }\left(x_{t},t\right)\right)+\sigma_{t}z$$

where σ is predefined according to $\sigma_{t}=\sqrt{\frac{1-{\bar{\alpha }}_{t-1}}{1-{\bar{\alpha }}_{t}}\beta_{t}}$ and z∼N(0,I).

### 2.4. Conditional Denoising Diffusion Probabilistic Model

The generation process of the DDPM is stochastic. To ensure that the DDPM produces the desired signal rather than a random signal from the learned distribution, it is necessary to incorporate a conditioner to guide the generation process. A conditional DDPM is a variant of the standard DDPM that employs a conditioner to control the generation process. Given a sample pair of a conditioner (denoted by *y*) and target data (denoted by $x_{0}$), the loss function of a conditional DDPM in training can be reformulated as follows:

(5) $$L\left(\theta \right)=E_{(x_{0},y),\epsilon ,t}{\left\|\epsilon -\epsilon_{\theta }\left(\sqrt{{\bar{\alpha }}_{t}}x_{0}+\sqrt{1-{\bar{\alpha }}_{t}}\epsilon ,t,y\right)\right\|}_{2}^{2}$$

## 3. Materials and Methods

### 3.1. Model Overview

**Figure 1:**
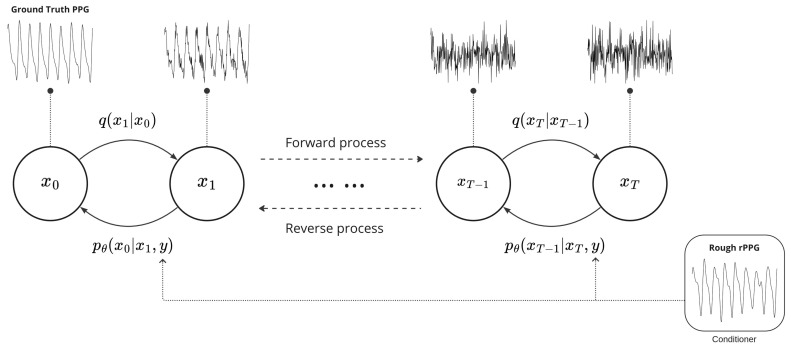
Conditional diffusion process with *T* steps. During the forward process, the ground truth PPG-signals denoted by $x_{0}$ are gradually transformed into a Gaussian noise $x_{T}$ with transition probability $q(x_{t}|x_{t-1})$. In the reverse process, the Gaussian noise is denoised to recover the ground truth PPG-signals with conditional transition probability $p_{\theta }\left(x_{t-1}|x_{t}\right)$. Rough rPPG-signals are utilized as conditioners to guide the reverse process to generate refined rPPG-signals resembling ground truth PPG-signals.

Figure 1 exemplifies the forward and reverse processes of DiffPhys, both of which proceed through *T* steps. The forward process starts from the ground truth PPG-signals (denoted by $x_{0}$) and gradually contaminates the ground truth PPG-signals by adding Gaussian noise in each step until the PPG-signals are transformed approximately into an isotropic Gaussian noise (denoised by $x_{T}$). This process is modeled with the Hidden Markov Model (HMM) with a transition probability of $q(x_{t}|x_{t-1})$ since each step is independent of the other steps. In the reverse process, DiffPhys begins from the isotropic Gaussian noise by denoising the noisy signals step by step until the ground truth PPG-signals are recovered. For each step *t*, a neural network is employed to denoise the noisy signal $x_{t+1}$ in the previous step to recover the less noisy signal $x_{t}$.

**Figure 2:**
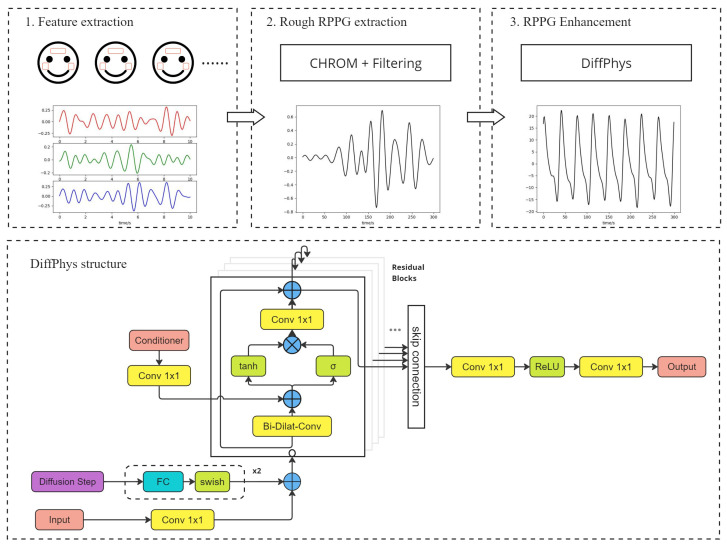
The flowchart at the top shows the pipeline of rPPG extraction and enhancement with DiffPhys. The bottom chart shows the network structure of DiffPhys. It should be noted that the bottom chart exemplifies one step of the reverse process of DiffPhys.

The pipeline of the rPPG extraction and enhancement is illustrated in Figure 2. Given the video of the subject, the regions of interest (ROIs) within each frame are located using face detection algorithms. Pixel values within each ROI are then averaged to output red, green, and blue (RGB) time series. The RGB time series are then processed by conventional rPPG extraction algorithms (CHROM is used here as an example) to produce rough rPPG-signals which are then filtered by bandpass filters to eliminate unrelated frequency elements. The rough rPPG-signals reveal periodicity that corresponds to the heart rate frequency but are not satisfying compared to the ground truth PPG-signals. After being processed by DiffPhys, the rPPG-signals closely resemble the ground truth PPG-signals, which shows the necessity of rPPG enhancement. The enhanced rPPG-signals can be utilized to estimate vital signs such as HR and HRV.

### 3.2. Network Structure

An overview of the neural network structure is illustrated at the bottom of Figure 2. We utilize DiffWave [20] as the backbone architecture. The network comprises a cascade of residual network blocks [21] where an identity map is applied to the input and the addend of the output. Each block is based on a bidirectional dilated convolution layer with different dilation steps, enabling multi-scale feature extraction from the input. The subsequent multiplication of the tanh and σ layers jointly produces periodic sinusoidal signals akin to rPPG-signals. The output of the residual block is fed into the subsequent residual block and the final output layers (conv1×1+ReLU+conv1×1) by skip connection. Consequently, the final output layers aggregate the output of all the residual blocks and generate the denoised signal $x_{t}$ as the input to the next step in the reverse process. This design allows for the efficient and robust extraction of rPPG-signals, leveraging the multi-scale features captured by the dilated convolutions and the periodic nature of the target signals.

Like the conventional conditional diffusion model, the input to DiffPhys comprises the input signal from previous step $x_{t+1}$, the position embedding of the step *t*, and the conditioner *y* (which is the rough rPPG-signals in this case). The input signal and step embedding are added up after being processed by different layers, as shown in the figure, and then fed into the residual block. The conditioner, on the other hand, is added to the output of the bidirectional dilated convolution layer after being processed by a convolution layer.

### 3.3. Training and Testing

The DiffPhys neural network is trained in a supervised manner where the input includes the noisy signal $x_{t+1}$ and the step *t*, and the output is the Gaussian noise that is added to $x_{t}$ at the *t* step of the forward process. The squared error between the estimated noise and the ground truth Gaussian noise serves as the loss function according to Equation (5). In the testing phase, a Gaussian noise is input to DiffPhys as the initial input $x_{T}$ of the reverse process. The initial Gaussian noise is gradually denoised by the neural network in each step until an enhanced rPPG signal is recovered. The denoising is implemented by feeding the noisy signal $x_{t+1}$ and step *t* into the neural network to estimate the Gaussian noise to be eliminated. The denoised signal $x_{t}$ is sampled according to Equation (4) and then fed into the neural network to estimate the next denoised signal $x_{t-1}$. After repeating for *T* steps, DiffPhys learns to generate rPPG-signals that resemble the ground truth PPG-signals from Gaussian noise. The structure of the neural network is detailed in Section 3.2.

However, since DiffPhys is trained on a large variety of rPPG-signals, the generated rPPG-signals can be quite random. To address this, rough rPPG-signals are used as conditioners (*y*) to guide the conditional transition probability in each step of the reverse process $p_{\theta }\left(x_{t-1}|x_{t},y\right)$. This ensures that the generated rPPG-signals closely resemble the ground truth PPG-signals.

### 3.4. Refined Reverse Process

Inspired by Choi et al. [22], we refine the reverse process by incorporating the conditioner as a bias to the standard reverse process. After DiffPhys generates the input to the next step $x_{t-1}$, an additional interpolation is conducted between the generated input and the conditioner *y*. For clarity, let ${\tilde{x}}_{t-1}$ and $x_{t-1}$ denote the generated observation at step t−1 before and after interpolation, respectively. The refined reverse process can be written as follows:

(6) $${\tilde{x}}_{t-1}=\frac{1}{\sqrt{{\bar{\alpha }}_{t}}}\left(x_{t}-\frac{\beta_{t}}{\sqrt{1-{\bar{\alpha }}_{t}}}\epsilon_{\theta }\left(x_{t},t,y\right)\right)+\sigma_{t}z$$

and

(7) $$x_{t-1}=\gamma_{t-1}{\tilde{x}}_{t-1}+\left(1-\gamma_{t-1}\right)y$$

where $\gamma_{t-1}$ is the hyper-parameter that regulates the refinement proportion. It starts from a large value when step *t* is large and decreases gradually as *t* approaches 0. Through this refinement, the search space in the reverse process is limited to the immediate space around the conditioner, leading to quicker convergence than the standard reverse process. In this case, we define $\gamma_{t-1}=\sqrt{{\bar{\alpha }}_{t-1}}$.

A key benefit of the refined reverse process is that it requires only minor adjustments to the reverse process without necessitating any further retraining of the neural network. The algorithm for the refined reverse process is given in Algorithm 1.
**Algorithm 1:** Refined reverse process.1:Sample $x_{T}∼N\left(0,I\right)$2:**for** t=T,T−1,⋯,1 **do**3:    Compute $\epsilon_{\theta }\left(x_{t},t,y\right)$4:    Sample z∼N(0,I)5:    Compute ${\tilde{x}}_{t-1}=\frac{1}{\sqrt{{\bar{\alpha }}_{t}}}\left(x_{t}-\frac{\beta_{t}}{\sqrt{1-{\bar{\alpha }}_{t}}}\epsilon_{\theta }\left(x_{t},t,y\right)\right)$6:    $\;+\sigma_{t}z$7:    Compute $x_{t-1}=\gamma_{t-1}{\tilde{x}}_{t-1}+\left(1-\gamma_{t-1}\right)y$8:**end for**9:**return** $x_{0}$

### 3.5. Fast Sampling

Currently, the reverse process is a lengthy procedure that impedes real-time applications of the diffusion model. In light of this, Kong et al. [23] drew inspiration from the sampling algorithm proposed by Chen et al. [24] and observed that the most effective denoising steps in the reverse process typically occur near t=0. We employ this fast sampling algorithm and adapt it to the refined reverse process, enabling accelerated generation of the PPG-signals.

## 4. Experiment Setup

In our experimental evaluation, CHROM was chosen as the baseline method to generate rough rPPG-signals due to its widespread acceptance as a commonly used benchmark within the field. Since DiffPhys was combined with CHROM as a posterior enhancement tool, the performance of CHROM signals before and after being processed by DiffPhys was compared to validate the effectiveness of DiffPhys. It should be noted that DiffPhys is compatible with a broad range of rPPG extraction methods and can be used to improve the waveform rPPG-signals extracted by these models.

For deep-learning-based methods, we selected DeepPhys and PhysFormer due to their superior performance in rPPG extraction. These models were implemented using the rPPG-Toolbox [25] to represent non-generative approaches. Additionally, PulseGAN was employed as a representative of the generative model. Our main comparison method, PulseGAN, is a GAN-based model specifically designed to enhance the quality of rPPG-signals. For fairness, all the benchmarking methods took preprocessed facial video as input and predicted rPPG-signals for comparison, and the rPPG-signals predicted by CHROM were further processed by DiffPhys to obtain the enhanced rPPG-signals. In addition, we also report a vanilla version of DiffPhys that uses a standard reverse process without refinement to assess the impact of the refined reverse process in the ablation study.

To evaluate the efficacy of our proposed method, we conducted experiments using the UBFC-rPPG dataset and PURE dataset, including both within-database and cross-database experiments. In the within-database experiments, we employed a five-fold cross-validation strategy, dividing the subjects into five equal groups. The models were trained in four groups and tested with the remaining group. For the cross-database experiments, the models were trained on one dataset and then tested on the other dataset.

### 4.1. Dataset

- UBFC-rPPG: The UBFC-rPPG dataset (available at https://sites.google.com/view/ybenezeth/ubfcrppg (accessed on 3 April 2024)) [26] contains video recordings of 42 different subjects playing games in bright environments. The videos were recorded using a Logitech C920 HD Pro webcam at 30 frames per second with a resolution of 640×480 pixels in uncompressed 8-bit RGB format. A CMS50E transmission pulse oximeter was used to obtain ground truth data for PPG.
- PURE: The PURE dataset (available at https://www.tu-ilmenau.de/universitaet/fakultaeten/fakultaet-informatik-und-automatisierung/profil/institute-und-fachgebiete/institut-fuer-technische-informatik-und-ingenieurinformatik/fachgebiet-neuroinformatik-und-kognitive-robotik/data-sets-code/pulse-rate-detection-dataset-pure (accessed on 3 April 2024)) [27] includes recordings of 10 individuals (8 males, 2 females) who executed six distinct predefined head movements, yielding a total of 60 video sequences, with each sequence lasting one minute. The video data were recorded using an eco274CVGE camera which operated at a 30 Hz frame rate and produced videos at 640×480 pixels resolution utilizing a 4.8mm lens. Pulse waves and SpO_2_ levels were collected using a pulox CMS50E finger clip pulse oximeter at a sampling rate of 60 Hz.

### 4.2. Preprocessing

The preservation of rough rPPG-signals involved a series of systematic steps. Firstly, facial landmarks were detected using Mediapipe [28]. The forehead and cheeks were selected as the regions of interest (ROIs). RGB signals were obtained by spatially averaging the pixels within the ROIs. Subsequently, the rough rPPG-signals were derived using a conventional rPPG extraction algorithm. A third-order bandpass Butterworth filter with a cutoff frequency range of 0.7 Hz to 3 Hz was applied to eliminate irrelevant interference frequencies. The filtered signals were then divided into segments of length L=300 sample points, with a stride of 3 sample points for the PURE dataset and 5 sample points for the UBFC-rPPG dataset, as the UBFC-rPPG dataset contains a larger number of subjects than the PURE dataset.

To ensure that the heart rates (HRs) were uniformly distributed between 0.7 Hz and 3 Hz, we resampled the rPPG-signals and corresponding PPG-signals. The ratio between the target frequency and the original frequency varied within [0.4, 0.6, 0.8, 1.2, 1.4, 1.6, 1.8, 2.0]. Resampled signals with frequencies outside the range of 0.7 Hz to 3 Hz were omitted. After resampling, we obtained 42,483 samples for the PURE dataset and 40,593 samples for the UBFC-rPPG dataset.

### 4.3. Training Configuration

The number of residual blocks within DiffPhys was 12 because we found that increasing the number of residual blocks did not bring improvement to the performance but led to longer training and inference time. Each of the residual blocks contained a bidirectional dilated convolution layer with a kernel size of 3. The dilation factor started at 1 in the first block and doubled in the subsequent block until it reached 8, following a cycle that repeated every four blocks. This cycle, [1, 2, 4, 8], was repeated three times across the 12 residual blocks.

For training, the noise schedules were linearly spaced as $\beta_{t}\in \left[1e^{-4},5e^{-2}\right]$ according to [23]. The learning rate was $2e^{-4}$, and the batch size was 64. An Adam optimizer was employed, and the maximum step was $2e^{6}$ where the training error displayed convergence. These hyper-parameters were selected based on a grid search in log10 space. For sampling, the fast sampling noise schedules were [0.0001, 0.001, 0.01, 0.05, 0.2, 0.5] according to [24].

### 4.4. Metrics

The mean absolute error (MAE), root mean square error (RMSE), mean absolute percentage error (MAPE), and Pearson Correlation (PC) between the predicted rPPG-signals and the ground truth PPG-signals were employed for HR metrics. The HR was calculated by taking the reverse of the averaged inter-beat intervals (IBI). It is defined as follows:

(8) $$\mathrm{HR}=\frac{N}{\sum_{i=1}^{N}{\mathrm{IBI}}_{i}}\times 60$$

For HRV, we employed AVNN and SDNN, which were the average and standard deviation of the normal-to-normal (NN) intervals, respectively, for the rPPG-signals and PPG-signals [29], defined as follows:

(9) $$\mathrm{AVNN}=\frac{1}{N}\sum_{i=1}^{N}{\mathrm{RR}}_{i}$$

and

(10) $$\mathrm{SDNN}=\sqrt{\frac{1}{N-1}\sum_{i=1}^{T}\left({\mathrm{RR}}_{t}-\mathrm{AVNN}\right)}$$

where ${\mathrm{RR}}_{i}$ is the *t*-th R-R interval, and *N* is the total number of R-R intervals. We used the MAE of the AVNN and the SDNN between the predicted rPPG-signals and the reference PPG-signals as metrics.

The SNR of the rPPG signal is defined as the ratio between the power around the fundamental frequency (HR frequency) and its first harmonic that corresponds to the reference PPG signal and the remaining noise power in the spectrum:

(11) $${\mathrm{SNR}}_{\mathrm{rPPG}-\mathrm{signal}}\left(\mathrm{dB}\right)=20\log_{10}\frac{P_{f_{0}}+P_{f_{1}}}{P_{\mathrm{noise}}}$$

where $P_{f_{0}}$ and $P_{f_{1}}$ are the power around the fundamental frequency and its first harmonic, respectively.

## 5. Results

### 5.1. Within Database

Table 1 reports the results for the within-database experiment on the PURE dataset. Both PulseGAN and DiffPhys showed better performance compared to non-generative deep-learning-based methods. The rough rPPG-signals generated by CHROM were not as good as DeepPhys and PhysFormer, but, after being processed by PulseGAN or DiffPhys, the SNR and the accuracy of HR and HRV improved significantly. DiffPhys with the refined reverse process achieved the best performance compared to all other methods. Specifically, compared to the baseline CHROM method, DiffPhys improved the performance of the MAE, RMSE, MAPE, and Pearson Correlation of HR by 69.90%, 63.86%, 64.48%, and 25.00%, respectively. It also decreased the MAE of AVNN and SDNN by 65.79% and 49.98%, respectively, while enhancing the SNR by 88.22%. These results indicate that DiffPhys effectively improved the quality of rough rPPG-signals extracted by the CHROM method on the PURE dataset.

For the UBFC-rPPG dataset, the results are shown in Table 2. Both DeepPhys and PhysFormer achieved good performance in HR estimation, but their performance on HRV was not as satisfying, which we believe is due to the overlooking of periodic properties of rPPG-signals. On the other side, the generative models achieved top-2 performance across all metrics, which shows the effectiveness of enhancing the rPPG-signals. DiffPhys with the refined reverse process achieved the best performance across all metrics but was not as salient as the results on the PURE dataset. After being processed by DiffPhys, the MAE, RMSE, and MAPE of HR were reduced by 67.08%, 83.67%, and 65.93%, respectively, while the Pearson Correlation was increased by 10%. For HRV-related metrics, the MAE of AVNN and SDNN were reduced by 75.58% and 44.38%, respectively. Additionally, the enhancement in SNR reached 37.96%. The within-database experiments on both datasets illustrate the efficacy of DiffPhys in improving the SNR of rPPG-signals.

### 5.2. Cross-Database

The results of the evaluation comparing various methods trained on the UBFC-rPPG dataset and tested on the PURE dataset are detailed in Table 3, indicating that DiffPhys significantly outperformed the baseline CHROM method. Specifically, DiffPhys achieved reductions in MAE, RMSE, and MAPE of 20.41%, 54.25%, and 21.43%, respectively, while enhancing the Pearson Correlation by 14.29%. The improvements were even more pronounced in HRV-related metrics, with reductions of 28.96% in AVNN MAE and 34.95% in SDNN MAE. Additionally, the SNR saw a substantial increase of 69.91%.

The results from training on the PURE dataset and testing on the UBFC-rPPG dataset, as depicted in Table 4, show that DiffPhys consistently outperformed other methods across all metrics. DiffPhys significantly enhanced the accuracy of HR estimation, achieving reductions in MAE, RMSE, and MAPE of 65.20%, 78.76%, and 64.04%, respectively. Additionally, it improved the Pearson Correlation by 10%. In terms of HRV metrics, DiffPhys reduced the MAE of AVNN and SDNN by 73.72% and 38.46%, respectively. Moreover, the SNR experienced an improvement of 40.72%. These results from both cross-database experiments underscore the exceptional generalization capability of DiffPhys in enhancing the SNR of rPPG-signals.

## 6. Discussion

### 6.1. Within Database

From the results of the within-database experiments on both the PURE and the UBFC-rPPG dataset, it can be seen that DiffPhys with the refined reverse process showed the best performance across all the metrics in HR and HRV estimations.

**Figure 3:**
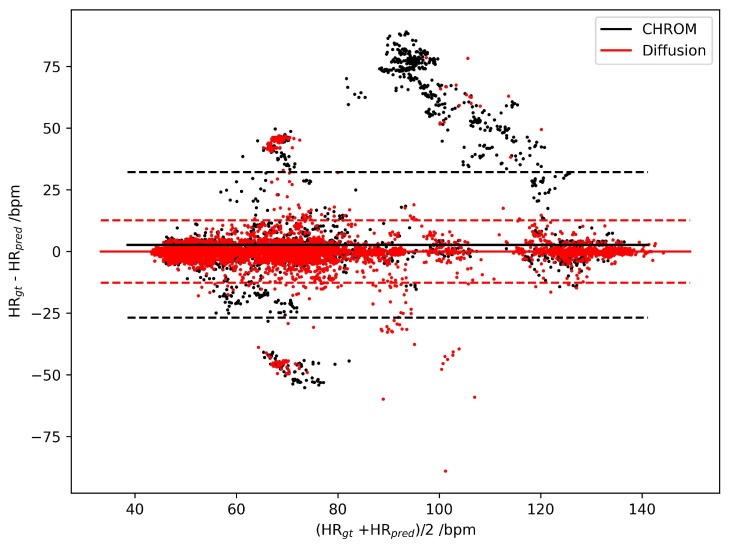
Bland–Altman Plots of HR estimation errors for CHROM (black dots) and DiffPhys (red dots) on the PURE dataset. Solid lines represent the mean of the HR estimation error distribution; dashed lines are boundaries corresponding to mean±1.96std.

**Figure 4:**
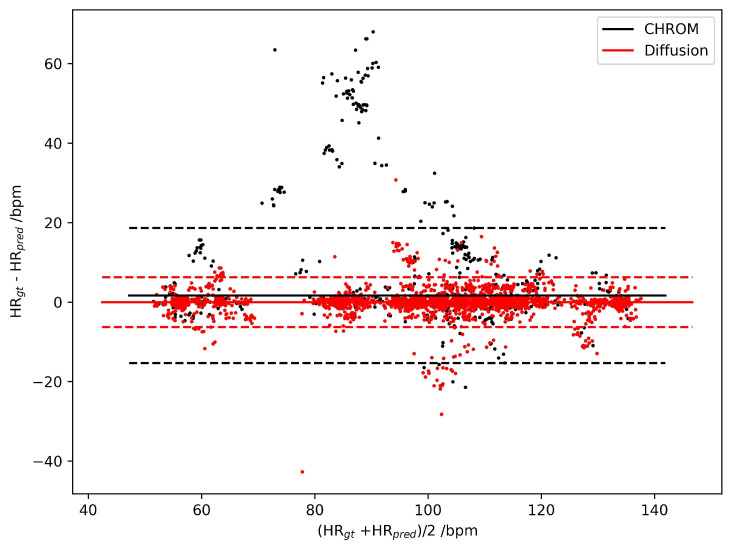
Bland–Altman Plots of HR estimation errors for CHROM (black dots) and DiffPhys (red dots) on the UBFC-rPPG dataset. Solid lines represent the mean of the HR estimation error distribution; dashed lines are boundaries corresponding to mean±1.96std.

For detailed evaluation, we illustrate the Bland–Altman Plots for the HR estimation error of CHROM and DiffPhys on the PURE (Figure 3) and UBFC-rPPG datasets (Figure 4). As shown in the figures, the difference between the ground truth HR and the HR estimated by DiffPhys was closer to 0 compared to CHROM in both cases. By conducting the Mann–Whitney U test on the absolute HR estimation error, we obtained $p=4.8e^{-14}$ for the PURE dataset and $p=9.1e^{-7}$ for the UBFC-rPPG dataset, indicating that the HR estimation error was significantly reduced after being processed by DiffPhys. The statistics demonstrate the effectiveness of DiffPhys as a posterior tool to enhance the SNR of rPPG-signals and the accuracy of the corresponding vital signs.

### 6.2. Cross-Database

The results from both cross-database experiments demonstrate the superior generalization capability of DiffPhys. This is particularly important in physiological signal analysis, where the model’s ability to perform well across different datasets is crucial. The substantial improvements in HR and HRV metrics and the enhanced SNR underscore DiffPhys’s robustness and reliability in diverse conditions.

Notably, most deep-learning-based methods underperformed relative to the CHROM method in the cross-dataset experiments with training on the UBFC-rPPG dataset and testing on the PURE dataset. This underperformance can be attributed to the greater diversity and challenges presented by the PURE dataset, which includes various subject motions. Despite this, all methods generally showed a decline in performance; however, DiffPhys consistently exceeded other performances across all metrics. This is a testament to its capability to effectively encode the prior distribution of a PPG signal, significantly boosting its robustness against variations in input rPPG-signals.

**Figure 5:**
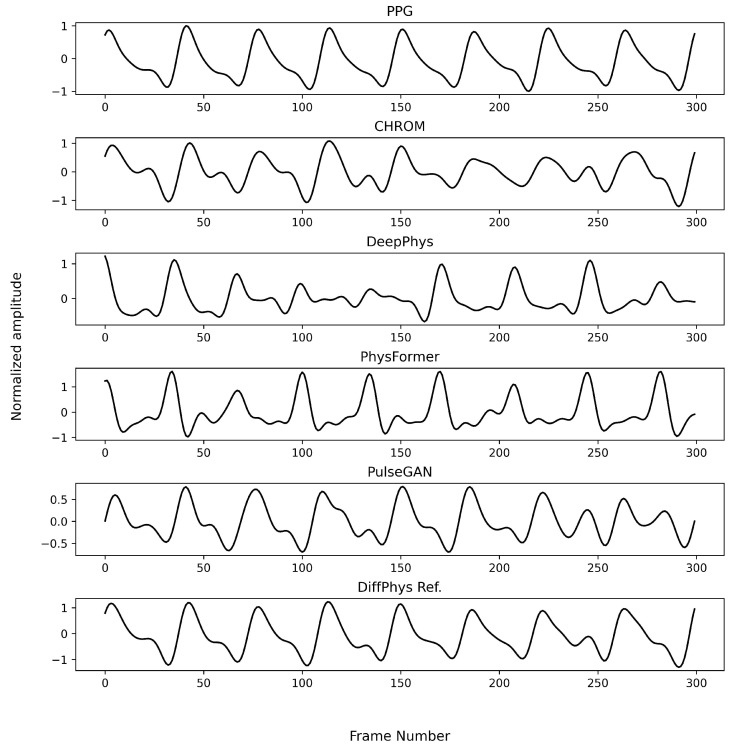
Enhanced rPPG-signals by different methods trained on the PURE dataset for a randomly selected example in the PURE dataset (within database). The first row is the ground truth PPG; the second row is the CHROM signal which is also the input signal to PulseGAN and DiffPhys models.

**Figure 6:**
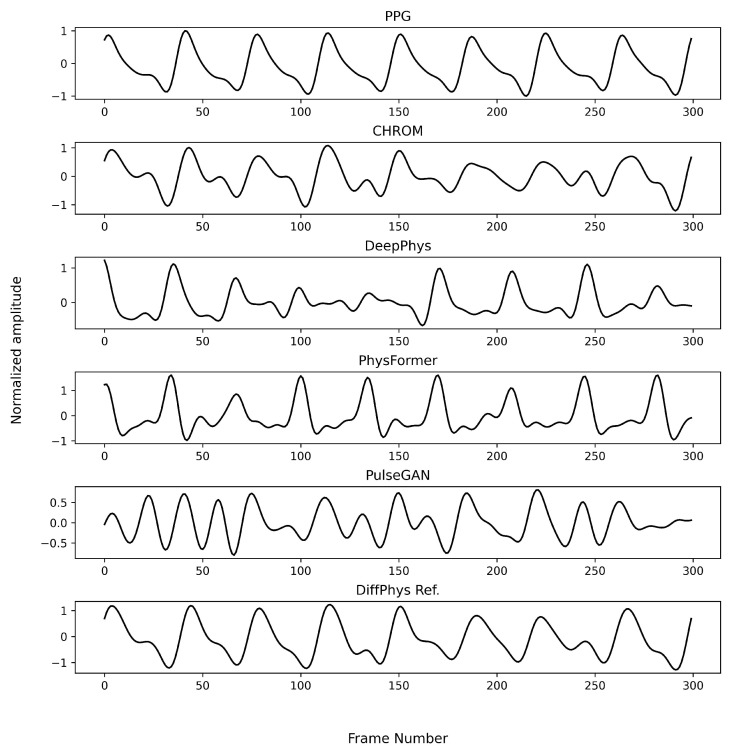
Enhanced rPPG-signals by different methods trained on the UBFC-rPPG dataset for a randomly selected example in the PURE dataset (cross-database).

To visualize the output of different benchmarking methods, we randomly selected a sample video from the PURE dataset and fed it into each benchmarking method trained on either the PURE dataset (which corresponds to the within-database experiment) or the UBFC-rPPG dataset (which corresponds to the cross-database experiment)The results for the within-database experiment are illustrated in Figure 5. All the methods produced rPPG-signals closely resembling the ground truth PPG-signals except for the non-generative methods, which showed minor variation. When it comes to the cross-database experiment as illustrated in Figure 6, the rPPG-signals produced by DeepPhys and PhysFormer still showed similar periodicity as the reference PPG-signal, while PulseGAN produced signals with different base frequency. In contrast, the rPPG-signals generated by DiffPhys maintained a higher fidelity to the ground truth, showing minimal degradation. This demonstrates the superior robustness and adaptability of DiffPhys in handling variations between different datasets.

### 6.3. Ablation Study

To assess the effect of the refined reverse process, we conducted an ablation study by training a DiffPhys model without the refined reverse process. The results, as presented in Table 5, indicate that the model incorporating the refined reverse process consistently surpassed the version without it in all metrics across various experiments. This finding underscores the effectiveness of the refined reverse process, highlighting its contribution to the overall performance enhancement of the DiffPhys model.

### 6.4. Real-World Applicability

In the experiments, the training time was about 34.5 ms for each sample with a duration of 10 s, and the inference time was about 38.6 ms with the help of fast sampling. The results demonstrate that DiffPhys is fast enough for real-world application.

Deploying DiffPhys in real-world scenarios involves handling a variety of conditions, such as different lighting environments, makeups, and facial expressions, which can impact the quality of rPPG-signals. To address these challenges, it is crucial to test and optimize DiffPhys under diverse conditions since the DiffPhys is highly dependent on the quality of rough rPPG-signals. Additionally, the model’s performance across different demographic groups, including variations in skin tone, age, and physiological characteristics, must be thoroughly evaluated. Training datasets should be diverse and representative of the broader population to mitigate any potential biases and ensure the model’s robustness.

## 7. Conclusions

In this work, we introduced DiffPhys, an innovative deep generative model rooted in the conditional DDPM designed to enhance the SNR of rPPG-signals. DiffPhys leverages rough rPPG-signals as conditioners to guide the reverse process, generating refined rPPG-signals with a higher SNR, as reflected in more accurate HR and HRV estimations. Experimental results demonstrated the competitive performance of DiffPhys in enhancing the SNR of rPPG-signals compared to other methods and its generalization ability on unseen samples in cross-database scenarios. The ablation study further proved the effectiveness of the refined reverse process in fine-tuning the performance of DiffPhys. DiffPhys provides a solution to enhance the SNR of rPPG-signals and can be easily integrated with other methods. This model can further improve the application of photoplethysmography in monitoring cardiovascular health based on facial videos.

Future efforts will concentrate on enhancing the performance of DiffPhys under more challenging circumstances and evaluating its capability to measure other vital signs.

## Figures and Tables

**Table 1:** Results for different methods on the PURE dataset. The best performance in each metric is bolded.

	HR (bpm)				HRV (ms)		
**Method**	**MAE**	**RMSE**	**MAPE**	**Pearson**	**AVNN**	**SDNN**	**SNR (dB)**
CHROM	4.85	16.27	5.18	0.72	40.19	42.18	5.35
DeepPhys	3.96	12.95	4.38	0.76	25.55	41.20	6.32
PhysFormer	4.36	13.00	5.60	0.76	24.83	29.11	6.31
PulseGAN	2.01	6.87	2.54	0.87	24.04	45.63	6.69
DiffPhys Ref. ^1^	**1.46**	**5.88**	**1.84**	**0.90**	**13.75**	**21.10**	**10.07**

^1^ DiffPhys Ref. is short for DiffPhys with refined reverse process.

**Table 2:** Results for different methods on UBFC-rPPG dataset. The best performance in each metric is bolded.

	HR (bpm)				HRV (ms)		
**Method**	**MAE**	**RMSE**	**MAPE**	**Pearson**	**AVNN**	**SDNN**	**SNR (dB)**
CHROM	3.19	9.98	3.17	0.90	29.11	24.21	4.92
DeepPhys	2.64	4.26	3.28	0.99	14.93	16.80	6.31
PhysFormer	2.83	6.43	3.32	0.96	11.24	23.05	6.01
PulseGAN	1.19	2.10	1.24	0.98	7.52	18.36	7.90
DiffPhys Ref.	**1.05**	**1.63**	**1.08**	**0.99**	**7.11**	**13.49**	**7.98**

**Table 3:** Results for different methods trained on UBFC-rPPG dataset and tested on PURE dataset. The best performance in each metric is bolded.

	HR (bpm)				HRV (ms)		
**Method**	**MAE**	**RMSE**	**MAPE**	**Pearson**	**AVNN**	**SDNN**	**SNR (dB)**
CHROM	4.85	16.27	5.18	0.72	40.19	42.18	5.35
DeepPhys	5.50	18.51	5.25	0.66	91.52	75.45	4.35
PhysFormer	13.19	24.57	24.41	0.47	170.37	47.13	2.16
PulseGAN	12.23	23.55	22.57	0.46	130.64	41.72	5.75
DiffPhys Ref.	**3.86**	**12.08**	**4.07**	**0.84**	**28.55**	**27.44**	**9.09**

**Table 4:** Results for different methods trained on PURE dataset and tested on UBFC-rPPG dataset. The best performance in each metric is bolded.

	HR (bpm)				HRV (ms)		
**Method**	**MAE**	**RMSE**	**MAPE**	**Pearson**	**AVNN**	**SDNN**	**SNR (dB)**
CHROM	3.19	9.98	3.17	0.90	29.11	24.21	4.92
DeepPhys	1.21	2.90	1.42	0.98	23.69	19.05	5.73
PhysFormer	1.69	6.64	1.70	0.93	14.65	19.30	3.44
PulseGAN	2.09	4.42	2.23	0.97	14.88	24.34	7.63
DiffPhys Ref.	**1.11**	**2.12**	**1.14**	**0.99**	**7.65**	**14.93**	**8.30**

**Table 5:** Ablation study for the effect of the refined reverse process on different datasets. P, PURE dataset; U, UBFC-rPPG dataset. The dataset on the left side of the arrow is the training dataset; the dataset on the right side of the arrow is the testing dataset. w/o Ref. and w/Ref. refer to DiffPhys without and with the refined reverse process, respectively. The best performance in each metric is bolded.

Setup		HR (bpm)				HRV (ms)		
**Dataset**	**Method**	**MAE**	**RMSE**	**MAPE**	**Pearson**	**AVNN**	**SDNN**	**SNR (dB)**
P→P	w/o Ref.	2.20	6.22	3.04	0.89	24.02	31.41	6.55
	w/Ref.	**1.46**	**5.88**	**1.84**	**0.90**	**13.75**	**21.10**	**10.07**
U→U	w/o Ref.	1.45	3.72	1.52	0.98	10.95	15.45	5.23
	w/Ref.	**1.05**	**1.63**	**1.08**	**0.99**	**7.11**	**13.49**	**7.98**
U→P	w/o Ref.	3.87	13.83	5.07	0.79	34.83	39.11	6.25
	w/Ref.	**3.86**	**12.08**	**4.07**	**0.84**	**28.55**	**27.44**	**9.09**
P→U	w/o Ref.	1.65	4.87	1.75	0.97	13.27	15.88	5.58
	w/Ref.	**1.11**	**2.12**	**1.14**	**0.99**	**7.65**	**14.93**	**8.30**

## Data Availability

Not applicable.
